# Impact of US industry payment disclosure laws on payments to surgeons: a natural experiment

**DOI:** 10.1186/s41073-019-0087-1

**Published:** 2020-01-03

**Authors:** Taeho Greg Rhee, Tijana Stanic, Joseph S. Ross

**Affiliations:** 1 Department of Public Health Sciences, School of Medicine, University of Connecticut, Farmington, CT USA; 2 Department of Psychiatry, Yale University School of Medicine, New Haven, CT USA; 3 VISN 1 New England Mental Illness, Research, Education, and Clinical Center (MIRECC), VA Connecticut Healthcare System, West Haven, CT USA; 4 Yale Center for Outcomes Research and Evaluation (CORE), Yale-New Haven Hospital, New Haven, CT USA; 5 Medical Practice Evaluation Center, Massachusetts General Hospital, Boston, MA USA; 6 Section of General Internal Medicine and the National Clinician Scholars Program, Department of Internal Medicine, Yale University School of Medicine, New Haven, CT USA; 7 Department of Health Policy and Management, Yale University School of Public Health, New Haven, CT USA

**Keywords:** Orthopedic, Pharmaceutical, Open payment, Physician Payment Sunshine Act, Centers for Medicare and Medicaid Services

## Abstract

**Objectives:**

To compare changes in the number and amount of payments received by orthopedic and non-orthopedic surgeons from industry between 2014 and 2017.

**Methods:**

Using the Centers for Medicare and Medicaid Services (CMS) Open Payment database from 2014 to 2017, we conducted a retrospective cohort study of industry payments to surgeons, including general payments and research payments.

**Results:**

Among orthopedic surgeons, the total number of general payments decreased from 248,698 in 2014 to 241,966 in 2017, but their total value increased from $97.1 million in 2014 to $110.2 million in 2017. Among non-orthopedic surgeons, the total number decreased from 604,884 in 2014 to 582,490 in 2017, while the total value remained stable at approximately $159 million. Between 2014 and 2017, there was a differential increase in the median number of general payments received by non-orthopedic when compared to orthopedic surgeons (incidence rate ratio, 1.09; 95% CI, 1.08–1.09; *p* < 0.001), but a differential decline in the median value of general payments (− 8.9%; 95% CI, − 9.5%, − 8.4%; *p* < 0.001). Findings were consistent when stratified by nature of payment. In contrast, between 2014 and 2017, there was neither a differential change in the median number nor median value of research payments received by non-orthopedics.

**Conclusion:**

Examination of a natural experiment of prior public disclosure of payments to orthopedic surgeons suggests that the Physician Payment Sunshine Act was associated with an increase in the number, but a decline in the value, of general payments received by non-orthopedic surgeons, but not on research payments received.

## Introduction

Interactions between physicians and teaching hospitals and the pharmaceutical and medical device industries can benefit patients when they are primarily related to bona fide basic and clinical research to improve patient care. However, in contrast to many other professions, such as education and law, the medical profession allows payments from a company to an individual physician who decides whether and how often to use products produced by the company. Yet, as the fields of clinical medicine, research, and marketing matured, there was a growing concern over the influence of industry on the medical profession.

For instance, one study estimated that, on average, physicians in the late 1990s met with industry representatives four times a month and residents accepted six gifts per year from industry representatives [1]. A more recent survey determined that 94% of physicians reported some type of relationship with the pharmaceutical industry, about 80% of which involved receiving food in the workplace or drug samples, whereas 35% also reported receiving reimbursements for costs associated with professional meetings or continuing medical education, 28% for consulting, giving lectures, or enrolling patients in clinical trials [2]. Similar survey studies demonstrated that relationships between industry and academic institutions, including Chairs, Departments, and Institutional Review Boards, were widespread [3, 4].

The pervasiveness of these relationships between industry and the medical profession, and the potential for undue influence, were the subject of several articles, conferences, and books, including by preeminent editors of the most prestigious biomedical journals [5–7]. These concerns were best summarized in a 2009 report by the Institute of Medicine, which concluded that the primary goals of medicine, defined as improving health by providing beneficial care to patients, conducting valid research, and offering excellent medical education, are at risk of being compromised by the undue pursuit of financial gain or other secondary interests posed by conflicts of interest [8].

In response to these concerns, Section 6002 of the Affordable Care Act of 2010 established the Open Payments program [9]. Known as the Physician Payment Sunshine Act, this legislation mandated pharmaceutical and medical device manufacturers to begin reporting, as of August 2013, nearly all payments to physicians and teaching hospitals to the Centers for Medicare & Medicaid Services (CMS) [10]. Numerous studies have made use of data from the Open Payments program, primarily to characterize the number, amount, and nature of the payments that have been received by physicians and academic medical centers from industry, frequently with a focus on specific physician specialties or payment types [11–18]. From these studies, it remains clear that many thousands of physicians continue to receive payments from industry, some for research, some for advisory board and consultancy work, but predominantly in-kind payments for food, travel, honoraria, and gifts. The frequency of these payments remains a cause for concern, as still other studies have used the Open Payments program data to characterize the association between these payments from industry to physicians and prescribing among Medicare beneficiaries [19, 20], demonstrating that payments are associated with greater rates of prescribing of the marketed drugs.

Little is known as to whether the enactment of the Physician Payment Sunshine Act led to changes in payments received by physicians, a question that is difficult to answer because of the lack of pre-legislation-required payment disclosure data. Our objective was to take advantage of a natural experiment of prior public disclosure of payments to orthopedic surgeons, who receive among the highest number of payments from industry across all medical sub-specialties [19, 21, 22], to better understand whether and how this legislation impacted payments received by physicians.

A natural experiment is an empirical or observational study, in which the control and experimental variables of interest are not artificially manipulated by researchers but instead are allowed to be influenced by nature or factors outside of the researchers’ control [23]. The natural experiment is particularly useful allowing for the study of whether orthopedic surgeons and non-orthopedic surgeons responded differently, with respect to payments received, when the Physician Payment Sunshine Act was enacted. In 2007, the Department of Justice reached a settlement with five companies that accounted for nearly 95% of the hip and knee surgical implant market, requiring mandatory disclosure of any payment from these companies to any physician, who were primarily orthopedic surgeons [24]. Leveraging this prior exposure, since payments to orthopedic surgeons have been publicly reported for years before the Sunshine Act went into effect, we compared changes in the number and amount of payments received by orthopedic and non-orthopedic surgeons between 2014 and 2017 using the Open Payments program data. Our hypothesis was that, because of prior exposure to disclosure laws, orthopedic surgeons would have a lower number and amount of payments received from industry when compared to non-orthopedic surgeons. The study is expected to offer insights into the impact of the enactment of the Sunshine Act on payments received by physicians, including whether there were differential changes in payments received for general activities vs. research.

## Methods

### Data source

The Open Payments program (https://openpaymentsdata.cms.gov) now makes data available on payments to physicians and teaching hospitals in the USA from August 2013 through December 2017, including information on 53.0 million payment disclosures totaling $33.4 billion [25]. These data have been viewed extensively, via the CMS website that provides both individual search and direct download tools [26], as well as via the Dollars for Doc website maintained by ProPublica [27], an independent investigative journalism organization that collates and sorts the Open Payments program data.

Reporting encompasses all direct and indirect payments for research, consulting, and advisory board service, as well as in-kind payments, such as food, travel, and gifts [9]. While payments with a value of less than $10 are exempted, if the annual aggregate total from one company to one recipient exceeds $100, they must then be reported. Also required as part of reporting by the legislation: the value of any payment, the paying manufacturer, and all products associated with the payment. These disclosures are made available to the general public [9, 28]; the legislation requires that the information should be available on a public website that is searchable, clear and understandable, and able to be easily aggregated and downloaded.

In this study, we included payments for general services and research but excluded payments for (1) royalties and licenses and (2) reports of ownership or investments, because such payments are generally considered compensation for intellectual property, and therefore, these are non-discretionary payments that are unlikely to be affected by the Sunshine Act.

### Measures

#### Orthopedic and non-orthopedic surgeons

We created an indicator variable that specifies whether the physician receiving the payment was an orthopedic surgeon likely to have been subject to the Department of Justice settlement with companies manufacturing hip and knee implants, hereafter orthopedic surgeons, or a surgeon of any other specialty, hereafter non-orthopedic surgeons. Orthopedic surgeons consisted of those in general orthopedic surgery, as well as those subspecializing in adult reconstructive orthopedic surgery, orthopedic trauma, and sports medicine, all of which may have financial engagements with hip and knee implant manufacturers. Non-orthopedic surgeons unlikely to have financial engagements with hip and knee implant manufacturers consisted of the following specialties: colon and rectal surgery; neurological surgery; foot and ankle surgery; hand surgery; orthopedic surgery of the spine; pediatric orthopedic surgery; otolaryngology and all sub-categories (i.e., facial plastic surgery, otolaryngic allergy, otology and neurotology, pediatric otolaryngology, plastic surgery within the head and neck, and sleep medicine); plastic surgery and its two sub-categories (i.e., plastic surgery within the head and neck, and surgery of the hand); and general surgery with its 10 sub-categories (i.e., hospice and palliative medicine, pediatric surgery, plastic and reconstructive surgery, surgery of the hand, critical care, surgical oncology, trauma surgery, vascular surgery, thoracic surgery (cardiothoracic vascular surgery), and transplant surgery).

#### Payments

Each payment was linked to a physician using a unique physician identification number. We created a count variable (continuous) for the total number of payments per physician, and the total value of payments (in US dollars) per physician. For all general payments, we created a binary variable to indicate whether the nature of payments was in the form of food and beverages, or some other form (i.e., speaker fees for education lectures, consulting fees, education, entertainment, honoraria, gifts, journal article reprints or textbooks, travel and lodging, research support, grants, charitable donations in lieu of payment, fees for space rental or use of facility, and others). We differentiated food and beverage payments from all other general payments because food and beverage payments make up nearly 90% of all payments, are considered by physicians to be “less worrisome” than other types of exchanges of value [29], and never involve payment in exchange for actual work or service. Thus, the final categories were based on whether physicians received payments (1) as in-kind gifts (e.g., food and beverage), (2) to act on behalf of companies (e.g., speaker fees or consulting fees), or (3) for research purposes.

### Analytical plans

First, we described the total number of physicians, total number of payments, median number of payments with inter-quartile range, total value of payments in US dollar, and median value of payments in US dollar by surgeon type (i.e., orthopedic vs. non-orthopedic) and by year for general payments and research payments received. For general payments, we also conducted a stratified analysis by the nature of payment (i.e., food and beverages vs. others).

Second, to test the hypothesis that, after enactment of the Sunshine Act, non-orthopedic surgeons will have received fewer payments from industry, and for lesser values, when compared with those orthopedic surgeons who were likely to have been previously exposed to “public transparency” of payment disclosures through the Department of Justice Settlement, we performed interaction analyses by year (2014 vs. 2017) and surgeon type (orthopedic vs. non-orthopedic) on the two outcomes of interest: median number of payments and median value of payments in US dollar.

In the regression models, the median number of payments was considered a count variable, such that a Poisson regression analysis was performed. The median value of payments in US dollars, on the other hand, was right-skewed, and therefore, was logarithm-transformed before running a regression analysis to correctly specify the functional form. We performed all analyses separately for both general and research payments. A stratified analysis was further conducted by the nature of payments (i.e., food and beverages, or others). All analyses were conducted using the Stata 15.1 MP/6-Core [30].

## Results

### General payments

Table 1 summarizes general payments received by orthopedic and non-orthopedic surgeons from 2014 to 2017. In 2014, 248,698 general payments were received by 21,685 orthopedic surgeons, totaling $97,063,276.39. In 2017, 241,966 general payments were received by 21,577 orthopedic surgeons, totaling $110,220,761.71. When stratified by the nature of payments, at least 70% of total number of payments were accounted for by food and beverages among those received by both orthopedic and non-orthopedic surgeons in 2014 and 2017. Among orthopedic surgeons, the median value of payments made in the form of food and beverages increased from $158.21 (IQR $56.69, $411.23) in 2014 to $171.50 (IQR $61.02, $429.39), and the median value of payments made in other forms increased from $1000.00 (IQR $264.55, $3381.03) in 2014 to $1510.19 (IQR 594.98, $4940.34). Similar patterns were found among non-orthopedic surgeons.

**Table 1:** General payments received by orthopedic and non-orthopedic surgeons, including total value, total number, and median value (inter-quartile range (IQR)) per capita, stratified by payment type, 2014–2017

	Total number of physicians	Total number of payments	Median number of payments (IQR)	Total value of payments, US $	Median value of payments, $ (IQR)
Orthopedic surgeons					
Overall payments					
2014	21,685	248,698	5 (2–13)	$97,063,276.39	$246.71 ($67.45–$1290.61)
2015	21,521	245,935	5 (2–13)	$97,888,851.41	$254.09 ($69.12–$1408.72)
2016	20,690	254,830	5 (2–15)	$112,789,015.27	$303.88 ($80.24–$1776.13)
2017	21,577	241,966	5 (2–13)	$110,220,761.71	$301.08 ($81.94–$1714.61)
Food and beverages					
2014	17,189	177,316	4 (2–12)	$7,455,902.20	$158.21 ($56.69–$411.23)
2015	17,470	173,938	4 (1–11)	$7,565,086.02	$159.42 ($55.00–$416.11)
2016	16,553	177,890	4 (1–13)	$7,834,719.11	$175.88 ($61.76–$458.98)
2017	17,302	169,440	4 (1–12)	$7,837,407.12	$171.50 ($61.02–$429.39)
Other sources					
2014	4393	71,382	7 (2–18)	$89,607,374.19	$1000.00 ($264.55–$3381.03)
2015	4051	71,997	9 (3–21)	$90,323,765.39	$1166.71 ($389.52–$4000.00)
2016	4137	76,940	10 (4–22)	$104,954,296.16	$1483.99 ($499.98–$5250.00)
2017	4275	72,526	9 (3–19)	$102,383,354.60	$1510.19 ($594.98–$4940.34)
Non-orthopedic surgeons					
Overall payments					
2014	60,164	604,884	4 (1–11)	$159,424,078.85	$170.63 ($51.60 - $774.88)
2015	59,167	610,441	4 (1–11)	$155,953,207.62	$185.93 ($52.50–$920.10)
2016	62,424	617,390	4 (1–11)	$161,997,987.22	$175.32 ($54.18–$886.37)
2017	56,899	582,490	4 (1–19)	$159,232,117.91	$188.25 ($57.74–$964.48)
Food and beverages					
2014	52,308	477,413	4 (1–10)	$19,974,145.33	$141.26 ($48.17–$369.92)
2015	51,375	480,997	4 (1–10)	$21,250,207.93	$145.80 ($46.86–$401.70)
2016	54,010	490,306	3 (1–10)	$22,553,812.03	$140.85 ($48.80–$394.76)
2017	49,718	457,736	3 (1–10)	$21,261,169.59	$147.61 ($51.52–$396.73)
Other sources					
2014	7822	127,471	7 (2–18)	$139,449,933.52	$1023.66 ($235.58–$3750.00)
2015	7792	129,444	7 (2–18)	$134,702,999.68	$1071.71 ($331.01–$3472.50)
2016	7414	127,084	8 (3–18)	$139,444,175.20	$1183.30 ($416.44–$4020.81)
2017	7181	124,754	8 (3–20)	$137,970,948.33	$1250.14 ($486.66–$4460.53)

Source: Open payments program data from the Centers for Medicare and Medicaid Services

### Research payments

Table 2 presents research payments received by orthopedic and non-orthopedic surgeons between 2014 and 2017. In 2014, 667 research payments were received by 259 orthopedic surgeons, totaling $2,006,252.77. In 2017, 541 research payments were received by 163 orthopedic surgeons, totaling $1,990,745.51. In 2014, 2239 696 non-orthopedic surgeons received research payments, totaling $12,462,894.96. In 2017, 1591 research payments were received by 526 non-orthopedic surgeons, totaling $6,401,187.11.

**Table 2:** Research payments received by orthopedic and non-orthopedic surgeons, including total value, total number, and median value (inter-quartile range (IQR)) per capita, 2014–2017

	Total number of physicians	Total number of payments	Median number of payments (IQR)	Total value of payments, US $ (IQR)	Median value, $ (IQR)
Orthopedic surgeons					
2014	259	667	1 (1–3)	$2,006,252.77	$3307.98 ($782.00–$8850.00)
2015	241	1181	3 (1–5)	$3,402,017.62	$4500.00 ($1200.00–$13,000.00)
2016	248	1025	2 (1–4)	$2,748,382.09	$4575.00 ($1159.74–$13,225.00)
2017	163	541	2 (1–4)	$1,990,745.51	$4355.00 ($1638.75–$11,310.00)
Non-orthopedic surgeons					
2014	696	2239	2 (1–3)	$12,462,894.96	$2495.00 ($700.00–$9625.00)
2015	838	3544	1 (1–3)	$9,428,075.11	$1325.00 ($556.98–$6200.00)
2016	727	2387	1 (1–2)	$8,618,203.73	$1602.75 ($510.00–$7098.79)
2017	526	1591	1 (1–3)	$6,401,187.11	$1650.00 ($690.00–$6700.00)

Source: Open payments program data from the Centers for Medicare and Medicaid Services

### Differential impact of the Physician Payment Sunshine Act

Tables 3 and 4 present interaction effects by year (2014 vs. 2017) and surgeon type (orthopedic vs. non-orthopedic) on the median number of and value of general and research payments to determine whether there was a differential impact of the Physician Payment Sunshine Act on non-orthopedic surgeons. For any general payments, non-orthopedic surgeons experienced a 9% differential increase (incidence rate ratio [IRR] = 1.09, 95% CI 1.08, 1.09; *p* < 0.001) in the median number of payments from 2014 to 2017 when compared to orthopedic surgeons. Similar patterns were found when stratified by the nature of payments. For general payments in the form of food and beverages, non-orthopedic surgeons experienced a 8% differential increase (IRR = 1.08, 95% CI 1.07, 1.09; *p* < 0.001) in the median number of payments from 2014 to 2017 when compared to orthopedic surgeons, whereas for general payments made in other forms, there was an 11% differential increase (IRR = 1.11, 95% CI 1.09, 1.13; *p* < 0.001).

**Table 3:** Interaction effects of period (2014 vs. 2017) and physician specialty (orthopedic vs. non-orthopedic surgeons) on median number of payments, 2014 and 2017

	Incidence rate ratio	95% CI	*P*-value
General payment: any	1.09	1.08, 1.09	< 0.001
General payment: food and beverage	1.08	1.07, 1.09	< 0.001
General payment: other sources	1.11	1.09, 1.13	< 0.001
Research payment	–^a^	–	–

^a^Indicates that could not be estimated due to a fully null effect

Source: Open payments program data from the Centers for Medicare and Medicaid Services

**Table 4:** Interaction effects of period (2014 vs. 2017) and physician specialty (orthopedic vs. non-orthopedic surgeons) on median value of payments (US dollars), 2014 and 2017

	% Change per capita	95% CI	*p* value
General payment: any	− 8.9%	– 9.5%, − 8.4%	< 0.001
General payment: food and beverage	− 2.9%	− 3.5%, − 2.3%	< 0.001
General payment: other sources	− 21.3%	− 22.3%, − 20.3%	< 0.001
Research payment	− 13.6%	− 28.5%, 4.6%	0.13

Source: Open payments program data from the Centers for Medicare and Medicaid Services

In contrast, for general payments, non-orthopedic surgeons experienced an 8.9% differential decline (95% CI: − 9.5%, − 8.4%; *p* < 0.001) in the median value of general payments received from 2014 to 2017 when compared to orthopedic surgeons. Again, similar patterns were found when stratified by the nature of payments. For general payments in the form of food and beverages, non-orthopedic surgeons experienced a 2.9% differential decline (95% CI − 3.5%, − 2.3%; *p* < 0.001) in the median value of general payments received from 2014 to 2017 when compared to orthopedic surgeons, whereas for general payments made in other forms, there was a 21.3% differential decline (95% CI: − 22.3%, − 20.3%; *p* < 0.001).

For research payments, any differential change in the median number of payments received by non-orthopedic surgeons from 2014 to 2017 when compared to orthopedic surgeons could not be estimated due to a fully null effect. Moreover, there was no differential change in the median value of research payments received by non-orthopedic surgeons from 2014 to 2017 when compared to orthopedic surgeons (− 13.6%; 95% CI − 28.5%, 4.6%; *p* = 0.13).

## Discussion

Taking advantage of a natural experiment mandating public disclosure of payments to orthopedic surgeons made by hip and knee surgical implant manufacturers, required as part of 2007 Department of Justice settlement, we tested the impact of the Physician Payment Sunshine Act on the number and value of payments received by physicians from industry. Our findings suggest that mandatory public disclosure of payments from industry was associated with increases in the number of general payments received, predominantly in the form of food and beverages, but with decreases in the median value of the general payments received. However, we found that the Physician Payment Sunshine Act was not associated with a differential change on research payments received. These results suggest that public transparency of the financial relationships between industry and physicians may be successfully mitigating some of the potential for undue influence that has been a source of concern within the medical profession, without diminishing financial support for bona fide basic and clinical research that is most likely to benefit patients.

Our study should be considered in the context of several important limitations. First, our study design is imperfect, as we are attempting to examine the impact of legislation that is difficult to isolate because of the lack of pre-legislation-required payment disclosure data. The Department of Justice settlement offers a unique opportunity to evaluate the legislation through the lens of a natural experiment, as the settlement exposed many orthopedic surgeons to mandatory disclosure of any payment from five companies that accounted for nearly 95% of the hip and knee surgical implant market beginning in 2008 [24]. Our hypothesis was that certain orthopedic surgeons likely to have financial engagements with hip and knee implant manufacturers would have accommodated their behaviors to the public scrutiny that potentially accompanies public disclosure payments from industry by the time of Sunshine Act implementation in late 2013. In contrast, many other surgeons would be unlikely to have financial engagements with hip and knee implant manufacturers and thus have no exposure to such public scrutiny, but would otherwise be similar in clinical practice patterns and industry engagement, providing a potential control cohort. Future research may consider using state-specific data to characterize differential changes among physicians to better understand the impact of the Physician Payment Sunshine Act, as several states, including Minnesota, Vermont, and Massachusetts, required public disclosure of payments to physicians before the Act went into effect, although with slightly different reporting requirements [10, 31, 32].

Second, our study examines the first four full years of the Open Payments program, beginning in 2014, but we were unable to examine data prior to the legislation’s requirements going into effect, prohibiting a true pre-post comparison. It is quite possible that all physicians, surgeons included, would have already changed their behaviors, including receipt of financial payments from industry, in anticipation of public disclosure beginning in late 2013. However, any such changes would have been likely to bias our findings to the null, suggesting that any effect observed was likely a consequence of surgeons who had little to no exposure with public scrutiny of their financial relationships with industry gradually changing in response. Nevertheless, factors other than the Sunshine Act may also account for changes in industry payments received by surgeons over time, including years of active practice and changes in the medical products being marketed and promoted to orthopedic vs. non-orthopedic surgeons, leading to differential changes in payments made by certain companies vs. others. Additional analyses will need to examine trends over a longer period of time, allowing for the observation of additional changes in payments received as physicians accommodate to the public scrutiny associated with the Open Payments program.

Despite these limitations, our analyses offer early insight into the likely impact of the Physician Payment Sunshine Act. Some may find our analyses reassuring, as it suggests that public scrutiny was associated with fewer general payments received among non-orthopedic surgeons, potentially limiting the influence that is expected to derive from financial relationships between industry and physicians. Because general payments are often made for services associated with detailing and marketing, including food and beverages, attending educational programs (which may or may not be accredited by professional organizations), and other non-specific honoraria, these general payments are both discretionary and less likely to represent services that would be expected to benefit patients. In contrast, research payments, which were unchanged in number and value, are more likely to represent services that are not marketing-based, but instead are funds to support scientific activities expected to benefit patients and broader general knowledge.

## Conclusions

In conclusion, our study suggests that mandatory public disclosure of payments from industry was associated with increases in the number of general payments received, predominantly in the form of food and beverages, but with decreases in the median value of the general payments received and no changes in research payments received. These findings suggest that public transparency of the financial relationships between industry and physicians may be successfully mitigating some of the potential for undue influence that has been a source of concern within the medical profession, without diminishing financial support for bona fide basic and clinical research that is most likely to benefit patients.

## Data Availability

Data and materials are publicly available in the following website: https://www.cms.gov/openpayments.

## Abbreviations

CMS: Centers for Medicare and Medicaid Services
IQR: Interquartile range
IRR: Incidence rate ratio
